# A decade of increasing complexity in patients sustaining a hip fracture in England, Wales and Northern Ireland: a national cohort study using the National Hip Fracture Database dataset

**DOI:** 10.1093/ageing/afag228

**Published:** 2026-08-11

**Authors:** Richard James Holleyman, Thomas Baldock, Andre Charlett, Paul Baker, Celia L Gregson, Andrew Judge, David John Deehan

**Affiliations:** Translational and Clinical Research Institute, Newcastle University, Newcastle upon Tyne, UK; Modelling and Economics Department, UK Health Security Agency (UKHSA), London, England, UK; Health Education England North East, Waterfront 4 Goldcrest Way, Newburn Riverside, Newcastle upon Tyne, UK; Modelling and Economics Department, UK Health Security Agency (UKHSA), London, England, UK; Department of Trauma and Orthopaedics, South Tees Hospitals NHS Foundation Trust, Middlesbrough, UK; Department of Health Sciences, University of York, York, UK; Ageing Research Unit, University of Bristol, Bristol, UK; Older Person's Unit, Royal United Hospital Bath NHS Trust, Bath**,** UK; Musculoskeletal Research Unit, Bristol Medical School: Translational Health Sciences, University of Bristol, Bristol**,** UK; Translational and Clinical Research Institute, Newcastle University, Newcastle upon Tyne, UK; Department of Orthopaedics, Freeman Hospital, Newcastle upon Tyne Hospitals NHS Trust, Newcastle upon Tyne, UK

**Keywords:** national hip fracture database, mortality, incidence, hip fracture, comorbid, older people

## Abstract

**Background:**

Hip fractures (HF) are a major cause of morbidity and mortality and account for substantial health and social care costs. We report the incidence and characteristics of older patients (≥60 years) presenting with hip fractures (HFs) in England, Wales and Northern Ireland.

**Methods:**

We conducted a national cohort study of HF events recorded in the National Hip Fracture Database (NHFD) between May 2011 and September 2023. HF incidence was derived using sex and age-banded mid-year population estimates from the Office for National Statistics to calculate at-risk population incidence for the whole cohort and by sex and age strata.

**Results:**

735 436 HF events were included (71% female, mean age 82.9 years). Annual NHFD HF numbers increased across the study period from 55 832 to 63 341 (13.5%). However, overall incidence declined from 422.8 to 412.8 per 100 000 population. Divergence was observed between sexes, with falling incidence in females but rising in males. HF patients are presenting with greater systemic disease burden, with the proportion graded American Society of Anaesthesiologists ≥3 increasing in both females (66.4% to 80.4%) and males (73.4% to 84.3%).

**Conclusion:**

Despite modest declines in overall incidence in women, absolute HF numbers continue to rise. The divergent sex-specific trends and substantial increase in patient comorbidity represent important shifts in HF epidemiology with implications for service capacity, risk stratification and secondary prevention strategies. This mandates urgent realignment of care resources to minimise the impact on patient mortality and economic health burden.

## Key Points

- Overall incidence declined from 422.8 to 412.8 per 100 000.
- Divergence was observed between sexes, with falling incidence in females but rising in males.
- Annual hip fracture numbers have increased from 55 832 to 63 341 (13.5%).
- Annual cyclical variation was observed, with peak incidence occurring in winter months being 10%–20% greater than summer nadirs.
- HF patients are presenting with greater systemic disease burden, with the proportion graded American Society of Anaesthesiologists ≥3 increasing in both sexes.

## Introduction

Hip fractures (HFs) are a major cause of morbidity and mortality among older adults, and account for substantial health and social care costs in the United Kingdom. Previous research has documented temporal shifts in the incidence and characteristics of patients sustaining HFs, with the number of fractures increasing year-on-year [1], and the relative proportion of male patients also rising [2, 3]. There are limited recent data on comorbidity trends, with rising patient comorbidity levels documented across multiple European national registries [4, 5].

The above trends are likely to be influenced by numerous factors, including changes in life expectancy, population structure, fracture prevention programmes and wider improvements in public health and medical and surgical care. More recently, the arrival of the SARS-CoV-2 (COVID-19) pandemic in early 2020 had profound effects on society, with older adults being at increased risk of mortality both generally [6], and in the HF cohort [7]. The pandemic may therefore have led to additional shifts in the characteristics of patients presenting with HFs that may impact patient outcomes, including mortality. Consequently, quantifying such temporal trends is crucial as they may also influence HF outcomes, including mortality and other key performance indicators. To our knowledge, no prior studies have specifically examined these characteristic trends in England, Wales and Northern Ireland and much of the existing literature is now over a decade old [4, 5].

The aim of this study was to report the incidence and describe the characteristics of patients presenting with HFs in England, Wales and Northern Ireland using data from the National Hip Fracture Database (NHFD) and to explore to what extent these characteristics varied following the COVID-19 pandemic.

## Methods

### Study design, data source and study population

We conducted a national cohort study of cases recorded in the NHFD having presented with an HF between 1 May 2011 and 1 September 2023, which included the period of the COVID-19 pandemic. The NHFD captures data for all patients ≥60 years old presenting to hospitals in England, Wales and Northern Ireland with fractures of the proximal femur; these fractures are represented within the International Classification of Diseases 10 (ICD-10) using codes S72.0: fractured neck of femur, S72.1: pertrochanteric fracture and S72.2: subtrochanteric fracture. The cohort included all eligible patients regardless of subsequent management, encompassing both operatively and non-operatively treated fractures.

### Patient, fracture and surgical characteristics

Available NHFD variables were captured, including patient age and sex, fracture type, American Society of Anaesthesiologists (ASA) grade, pre-operative 10-point abbreviated mental test score (AMTS), nutritional status at presentation and residential and mobility status before admission. Malnutrition was recorded from assessments conducted by nursing staff using the malnutrition universal screening tool (MUST) score [8].

### Statistical analysis

Patient and injury characteristics were summarised by calendar year and stratified by sex. To calculate HF incidence, we used sex and age-banded mid-year population estimates for England, Wales and Northern Ireland from the Office for National Statistics (ONS) to calculate crude at-risk population incidence for the whole cohort and by sex and age strata [9]. Mid-year population estimates reported by ONS as at 30 June each year were linearly interpolated between years to calculate a continuous estimate of population size between years.

To investigate temporal and cyclical trends—including those that may have been acute or transient, for which annual aggregation might be insufficiently sensitive—HF counts and incidence were presented as a non-centred moving average over a retrospective 30-day period. To relate these trends with reference to the COVID-19 pandemic, we defined the ‘COVID-19 period’ as extending from 20^th^ March 2020 (the start of the UK lockdown) until 31^st^ March 2022 (the end of widespread community COVID-19 testing).

Missing data were reported for each variable explicitly and, where indicated, unrecorded or missing data were excluded from column percentage calculations.

### Study approvals

Approval for this study was granted by the UK Healthcare Quality Improvement Partnership in January 2024 (ref: HQIP286).

### Results

The study population included 735 436 NHFD HF events occurring between 1^st^ May 2011 and 2^nd^ September 2023 after data quality exclusions (see figure Appendix 1 in supplementary materials for study flow diagram). Across all cases, 71% were female, and the overall mean age was 82.9 years (females: 83.3 years; males: 81.6 years). Patient and injury characteristics by year and stratified by sex are shown in Table 1 and Table 2.

**Table 1:** Female patient characteristics by year.

	Year of presentation (n)												
Variable	2011	2012	2013	2014	2015	2016	2017	2018	2019	2020	2021	2022	2023
	−26 581	−41 136	−42 508	−42 379	−42 782	−42 499	−43 016	−42 890	−43 333	−41 817	−40 644	−44 172	−29 683
**Mean age (SD), 95%CI**	83.32 (8.2);	83.36 (8.2);	83.39 (8.3);	83.33 (8.4);	83.31 (8.4);	83.30 (8.4);	83.19 (8.6);	83.13 (8.6);	83.14 (8.5);	83.28 (8.5);	83.18 (8.6);	82.97 (8.6);	82.94 (8.6);
	83.22 to 83.4	83.28 to 83.4	83.31 to 83.5	83.25 to 83.4	83.23 to 83.4	83.22 to 83.4	83.11 to 83.3	83.05 to 83.2	83.06 to 83.2	83.20 to 83.4	83.10 to 83.3	82.89 to 83.0	82.84 to 83.0
**ASA Grade**													
ASA 1	578 (2.4)	870 (2.3)	890 (2.2)	971 (2.4)	882 (2.1)	857 (2.1)	827 (2.0)	767 (1.8)	641 (1.5)	543 (1.3)	516 (1.3)	521 (1.2)	346 (1.2)
ASA 2	7961 (32.9)	11 975 (31.3)	12 456 (30.8)	11 797 (29.1)	11 642 (28.4)	10 983 (26.7)	10 516 (25.2)	9 838 (23.6)	9330 (22.1)	7884 (19.5)	7414 (18.7)	7926 (18.5)	5199 (18.0)
ASA 3	13 103 (54.1)	21 266 (55.5)	22 263 (55.1)	22 761 (56.1)	23 211 (56.5)	23 490 (57.2)	23 872 (57.1)	24 011 (57.5)	24 600 (58.3)	24 551 (60.6)	24 253 (61.3)	26 459 (61.7)	17 898 (61.8)
ASA 4	2480 (10.2)	4017 (10.5)	4640 (11.5)	4887 (12.1)	5165 (12.6)	5601 (13.6)	6404 (15.3)	6951 (16.6)	7466 (17.7)	7421 (18.3)	7281 (18.4)	7878 (18.4)	5427 (18.8)
ASA 5	91 (0.38)	170 (0.44)	169 (0.42)	129 (0.32)	164 (0.40)	158 (0.38)	177 (0.42)	183 (0.44)	154 (0.37)	113 (0.28)	119 (0.30)	134 (0.31)	68 (0.23)
Not recorded^†^	2368	2838	2 090	1834	1718	1410	1220	1140	1142	1305	1061	1254	745
Residence^*^													
Own home/sheltered housing	19 730 (74.2)	30 581 (74.3)	31 983 (75.2)	32 222 (76.0)	32 773 (76.6)	32 757 (77.1)	33 326 (77.5)	33 601 (78.3)	34 019 (78.5)	32 810 (78.5)	32 422 (79.8)	35 261 (79.8)	23 566 (79.4)
Residential care	3638 (13.7)	5354 (13.0)	5212 (12.3)	5274 (12.4)	5446 (12.7)	5111 (12.0)	5243 (12.2)	4873 (11.4)	5006 (11.6)	4803 (11.5)	4363 (10.7)	4880 (11.0)	3348 (11.3)
Nursing care	1808 (6.8)	2994 (7.3)	3215 (7.6)	3277 (7.7)	3218 (7.5)	3193 (7.5)	3014 (7.0)	3026 (7.1)	2990 (6.9)	3125 (7.5)	2887 (7.1)	2967 (6.7)	2056 (6.9)
Hospital inpatient	983 (3.7)	1577 (3.8)	1547 (3.6)	1420 (3.4)	1243 (2.9)	1437 (3.4)	1432 (3.3)	1384 (3.2)	1315 (3.0)	1062 (2.5)	968 (2.4)	1049 (2.4)	710 (2.4)
Other	422 (1.6)	630 (1.5)	551 (1.3)	186 (0.44)	102 (0.24)	1 (0.00)	1 (0.00)	6 (0.01)	3 (0.01)	17 (0.04)	4 (0.01)	15 (0.03)	3 (0.01)
**Mobility**													
Freely mobile without aids	8090 (34.8)	12 163 (32.8)	12 904 (32.8)	14 736 (35.8)	15 497 (36.7)	15 479 (36.7)	15 519 (36.4)	15 634 (36.8)	15 918 (37.1)	15 231 (36.8)	15 024 (37.4)	16 750 (38.5)	11 348 (38.6)
Mobile outdoors with one or more aids or a frame	5242 (22.6)	8125 (21.9)	8603 (21.9)	13 409 (32.6)	15 415 (36.5)	15 167 (36.0)	15 558 (36.5)	15 612 (36.7)	15 679 (36.5)	14 961 (36.2)	14 065 (35.0)	15 999 (36.7)	10 569 (36.0)
Never goes outside without help or no functional mobility	9912 (42.6)	16 738 (45.2)	17 788 (45.3)	13 038 (31.7)	11 327 (26.8)	11 475 (27.2)	11 536 (27.1)	11 255 (26.5)	11 357 (26.4)	11 178 (27.0)	11 103 (27.6)	10 812 (24.8)	7476 (25.4)
Not recorded^†^	3337	4110	3213	1196	543	378	403	389	379	447	452	611	290
**Nutrition assessment**													
Normal nutrition	0 (0)	0 (0)	0 (0)	0 (0)	0 (0)	26 303 (71.8)	29 343 (71.1)	30 750 (73.2)	30 961 (72.8)	29 037 (71.9)	28 587 (72.7)	30 751 (72.7)	20 989 (72.9)
At risk of malnutrition	0 (0)	0 (0)	0 (0)	0 (0)	0 (0)	7933 (21.7)	8919 (21.6)	7816 (18.6)	8256 (19.4)	8186 (20.3)	7748 (19.7)	8212 (19.4)	5564 (19.3)
Malnourished	0 (0)	0 (0)	0 (0)	0 (0)	0 (0)	2391 (6.5)	2980 (7.2)	3445 (8.2)	3333 (7.8)	3142 (7.8)	2989 (7.6)	3349 (7.9)	2236 (7.8)
Not recorded^†^	26 581	41 136	42 508	42 379	42 782	5872	1774	879	783	1452	1320	1860	894
**AMTS on admission**													
10	7722 (29.1)	14 089 (34.2)	15 359 (36.1)	15 666 (37.0)	15 951 (37.3)	16 102 (37.9)	16 558 (38.5)	16 576 (38.6)	16 993 (39.2)	16 215 (38.8)	15 646 (38.5)	17 671 (40.0)	12 107 (40.8)
7 to 9	4017 (15.1)	9497 (23.1)	11 185 (26.3)	11 475 (27.1)	11 541 (27.0)	11 467 (27.0)	11 466 (26.7)	11 423 (26.6)	11 381 (26.3)	10 680 (25.5)	10 611 (26.1)	11 097 (25.1)	7250 (24.4)
0 to 6	5679 (21.4)	12 523 (30.4)	13 984 (32.9)	14 089 (33.2)	14 306 (33.4)	13 920 (32.8)	13 844 (32.2)	13 941 (32.5)	13 961 (32.2)	13 643 (32.6)	13 114 (32.3)	13 577 (30.7)	9018 (30.4)
Not recorded or patient refused	9163 (34.5)	5027 (12.2)	1980 (4.7)	1149 (2.7)	984 (2.3)	1010 (2.4)	1148 (2.7)	950 (2.2)	998 (2.3)	1279 (3.1)	1273 (3.1)	1827 (4.1)	1308 (4.4)
**Mode of presentation with hip fracture**													
Presented via A&E	25 126 (94.8)	38 951 (94.8)	40 410 (95.2)	40 395 (95.6)	40 944 (96.1)	40 971 (96.6)	41 576 (96.7)	41 501 (96.8)	42 016 (97.0)	40 737 (97.5)	39 654 (97.6)	43 039 (97.6)	28 971 (97.6)
Already an inpatient	0 (0)	0 (0)	0 (0)	0 (0)	1 (0.00)	1437 (3.4)	1432 (3.3)	1384 (3.2)	1315 (3.0)	1062 (2.5)	968 (2.4)	1049 (2.4)	710 (2.4)
Other presentation	1367 (5.2)	2130 (5.2)	2052 (4.8)	1869 (4.4)	1663 (3.9)	0 (0)	0 (0)	0 (0)	0 (0)	0 (0)	0 (0)	0 (0)	0 (0)
Not recorded^†^	88	55	46	115	174	91	8	5	2	18	22	84	2
**Hip fracture type**													
Displaced Intracapsular	12 739 (47.9)	19 676 (47.8)	20 835 (49.0)	20 869 (49.2)	20 897 (48.8)	20 925 (49.2)	21 137 (49.1)	21 029 (49.0)	21 741 (50.2)	21 239 (50.8)	20 957 (51.6)	23 009 (52.1)	15 566 (52.4)
Undisplaced Intracapsular	3069 (11.5)	4311 (10.5)	4102 (9.6)	4223 (10.0)	4314 (10.1)	3971 (9.3)	3949 (9.2)	3891 (9.1)	3326 (7.7)	2901 (6.9)	2862 (7.0)	3057 (6.9)	1866 (6.3)
Intertrochanteric	9333 (35.1)	14 731 (35.8)	15 083 (35.5)	14 716 (34.7)	14 902 (34.8)	15 073 (35.5)	15 450 (35.9)	15 635 (36.5)	16 039 (37.0)	15 279 (36.5)	14 609 (35.9)	15 732 (35.6)	10 675 (36.0)
Subtrochanteric	1440 (5.4)	2418 (5.9)	2488 (5.9)	2571 (6.1)	2669 (6.2)	2530 (6.0)	2480 (5.8)	2335 (5.4)	2227 (5.1)	2398 (5.7)	2216 (5.5)	2374 (5.4)	1576 (5.3)

Characteristics of female patients in the study cohort by year. †, Cases with unrecorded/missing data were excluded from column percentage calculations. 95%CI indicates the 95% confidence interval of the mean for given column. ASA, American Society of Anaesthesiologists. AMTS, Abbreviated Mental Test Score. Data for 2011 and 2023 include only partial years.

**Table 2:** Male patient characteristics by year.

	Year of presentation (n)												
Variable	2011	2012	2013	2014	2015	2016	2017	2018	2019	2020	2021	2022	2023
	−9255	−14 696	−16 262	−16 583	−17 001	−17 345	−17 792	−17 876	−18 116	−17 751	−17 548	−19 169	−12 602
**Mean age (SD), 95%CI**	81.37 (8.5);	81.52 (8.6);	81.64 (8.6);	81.66 (8.6);	81.55 (8.7);	81.56 (8.7);	81.51 (8.8);	81.53 (8.8);	81.68 (8.7);	81.85 (8.7);	81.58 (8.8);	81.43 (8.9);	81.54 (8.8);
	81.20 to 81.5	81.38 to 81.7	81.51 to 81.8	81.53 to 81.8	81.42 to 81.7	81.44 to 81.7	81.39 to 81.6	81.40 to 81.7	81.55 to 81.8	81.72 to 82.0	81.45 to 81.7	81.30 to 81.6	81.39 to 81.7
**ASA Grade**													
ASA 1	206 (2.5)	266 (2.0)	280 (1.8)	309 (2.0)	313 (1.9)	310 (1.9)	289 (1.7)	267 (1.5)	215 (1.2)	214 (1.3)	205 (1.2)	227 (1.2)	114 (0.93)
ASA 2	2144 (25.6)	3345 (24.6)	3611 (23.5)	3625 (22.9)	3525 (21.7)	3395 (20.4)	3367 (19.5)	3118 (18.0)	2988 (17.0)	2698 (15.8)	2601 (15.3)	2659 (14.4)	1685 (13.8)
ASA 3	4713 (56.3)	7793 (57.4)	8918 (57.9)	9134 (57.7)	9428 (58.0)	9712 (58.3)	9940 (57.7)	10 049 (57.9)	10 259 (58.3)	10 027 (58.6)	10 206 (60.0)	11 222 (60.6)	7591 (62.0)
ASA 4	1254 (15.0)	2088 (15.4)	2501 (16.2)	2684 (16.9)	2892 (17.8)	3130 (18.8)	3511 (20.4)	3791 (21.8)	4014 (22.8)	4053 (23.7)	3938 (23.1)	4299 (23.2)	2812 (23.0)
ASA 5	56 (0.67)	86 (0.63)	84 (0.55)	85 (0.54)	87 (0.54)	108 (0.65)	128 (0.74)	136 (0.78)	110 (0.63)	111 (0.65)	70 (0.41)	97 (0.52)	43 (0.35)
Not recorded^†^	882	1118	868	746	756	690	557	515	530	648	528	665	357
Residence*													
Own home/sheltered housing	7080 (76.5)	11 099 (75.5)	12 449 (76.6)	12 869 (77.6)	13 334 (78.4)	13 622 (78.5)	13 970 (78.5)	14 355 (80.3)	14 415 (79.6)	14 202 (80.0)	14 394 (82.0)	15 797 (82.4)	10 276 (81.5)
Residential care	891 (9.6)	1388 (9.4)	1404 (8.6)	1548 (9.3)	1596 (9.4)	1537 (8.9)	1623 (9.1)	1506 (8.4)	1533 (8.5)	1516 (8.5)	1369 (7.8)	1399 (7.3)	1037 (8.2)
Nursing care	573 (6.2)	931 (6.3)	1097 (6.7)	1141 (6.9)	1138 (6.7)	1175 (6.8)	1165 (6.5)	1148 (6.4)	1195 (6.6)	1187 (6.7)	1014 (5.8)	1102 (5.7)	748 (5.9)
Hospital inpatient	537 (5.8)	971 (6.6)	983 (6.0)	924 (5.6)	892 (5.2)	1011 (5.8)	1033 (5.8)	866 (4.8)	971 (5.4)	833 (4.7)	767 (4.4)	868 (4.5)	537 (4.3)
Other	174 (1.9)	307 (2.1)	329 (2.0)	101 (0.61)	41 (0.24)	0 (0)	1 (0.01)	1 (0.01)	2 (0.01)	13 (0.07)	4 (0.02)	3 (0.02)	4 (0.03)
**Mobility**													
Freely mobile without aids	2830 (34.8)	4462 (33.7)	5012 (33.2)	5606 (34.9)	6227 (37.1)	6311 (36.7)	6398 (36.3)	6539 (36.9)	6514 (36.3)	6370 (36.3)	6527 (37.6)	7309 (38.8)	4699 (37.7)
Mobile outdoors with one or more aids or a frame	2174 (26.8)	3568 (27.0)	4002 (26.5)	5960 (37.1)	6627 (39.5)	6712 (39.0)	7056 (40.1)	6884 (38.9)	7119 (39.6)	7029 (40.1)	6648 (38.3)	7485 (39.7)	4896 (39.3)
Never goes outside without help or no functional mobility	3121 (38.4)	5206 (39.3)	6060 (40.2)	4502 (28.0)	3912 (23.3)	4166 (24.2)	4152 (23.6)	4287 (24.2)	4332 (24.1)	4142 (23.6)	4185 (24.1)	4062 (21.5)	2866 (23.0)
Not recorded^†^	1130	1460	1188	515	235	156	186	166	151	210	188	313	141
**Nutrition assessment**													
Normal nutrition	0 (0)	0 (0)	0 (0)	0 (0)	0 (0)	11 179 (75.1)	12 784 (74.9)	13 608 (77.9)	13 867 (78.0)	13 048 (76.4)	13 232 (77.9)	14 294 (78.3)	9512 (78.0)
At risk of malnutrition	0 (0)	0 (0)	0 (0)	0 (0)	0 (0)	2899 (19.5)	3363 (19.7)	2774 (15.9)	2866 (16.1)	3014 (17.6)	2807 (16.5)	2927 (16.0)	1974 (16.2)
Malnourished	0 (0)	0 (0)	0 (0)	0 (0)	0 (0)	815 (5.5)	911 (5.3)	1094 (6.3)	1044 (5.9)	1018 (6.0)	936 (5.5)	1045 (5.7)	704 (5.8)
Not recorded^†^	9255	14 696	16 262	16 583	17 001	2452	734	400	339	671	573	903	412
**AMTS on admission**													
10	2851 (30.8)	5416 (36.9)	6496 (39.9)	6628 (40.0)	6963 (41.0)	7195 (41.5)	7436 (41.8)	7555 (42.3)	7646 (42.2)	7518 (42.4)	7486 (42.7)	8242 (43.0)	5539 (44.0)
7 to 9	1387 (15.0)	3387 (23.0)	4256 (26.2)	4502 (27.1)	4697 (27.6)	4733 (27.3)	4797 (27.0)	4793 (26.8)	4951 (27.3)	4645 (26.2)	4675 (26.6)	4950 (25.8)	3175 (25.2)
0 to 6	1665 (18.0)	3880 (26.4)	4606 (28.3)	4894 (29.5)	4841 (28.5)	4904 (28.3)	4968 (27.9)	5023 (28.1)	5057 (27.9)	4974 (28.0)	4772 (27.2)	5067 (26.4)	3249 (25.8)
Not recorded or patient refused	3352 (36.2)	2013 (13.7)	904 (5.6)	559 (3.4)	500 (2.9)	513 (3.0)	591 (3.3)	505 (2.8)	462 (2.6)	614 (3.5)	615 (3.5)	910 (4.7)	639 (5.1)
**Mode of presentation with hip fracture**													
Presented via A&E	8501 (92.2)	13 452 (91.7)	14 974 (92.2)	15 432 (93.3)	15 870 (93.8)	16 283 (94.2)	16 757 (94.2)	17 009 (95.2)	17 139 (94.6)	16 909 (95.3)	16 770 (95.6)	18 255 (95.5)	12 065 (95.7)
Already an inpatient	0 (0)	0 (0)	0 (0)	0 (0)	0 (0)	1011 (5.8)	1033 (5.8)	866 (4.8)	971 (5.4)	833 (4.7)	767 (4.4)	868 (4.5)	537 (4.3)
Other presentation	724 (7.8)	1221 (8.3)	1259 (7.8)	1110 (6.7)	1054 (6.2)	0 (0)	0 (0)	0 (0)	0 (0)	0 (0)	0 (0)	0 (0)	0 (0)
Not recorded^†^	30	23	29	41	77	51	2	1	6	9	11	46	0
**Hip fracture type**													
Displaced Intracapsular	4513 (48.8)	7218 (49.1)	8277 (50.9)	8374 (50.5)	8592 (50.5)	8911 (51.4)	9102 (51.2)	9316 (52.1)	9579 (52.9)	9536 (53.7)	9524 (54.3)	10 460 (54.6)	6931 (55.0)
Undisplaced Intracapsular	1028 (11.1)	1593 (10.8)	1634 (10.0)	1674 (10.1)	1747 (10.3)	1633 (9.4)	1647 (9.3)	1536 (8.6)	1408 (7.8)	1265 (7.1)	1280 (7.3)	1295 (6.8)	772 (6.1)
Intertrochanteric	3201 (34.6)	5076 (34.5)	5407 (33.2)	5556 (33.5)	5566 (32.7)	5793 (33.4)	6051 (34.0)	6005 (33.6)	6241 (34.5)	5922 (33.4)	5805 (33.1)	6436 (33.6)	4251 (33.7)
Subtrochanteric	513 (5.5)	809 (5.5)	944 (5.8)	979 (5.9)	1096 (6.4)	1008 (5.8)	992 (5.6)	1019 (5.7)	888 (4.9)	1028 (5.8)	939 (5.4)	978 (5.1)	648 (5.1)

Characteristics of male patients in the study cohort by year. †, Cases with unrecorded/missing data were excluded from column percentage calculations. 95%CI indicates the 95% confidence interval of the mean for given column. ASA, American Society of Anaesthesiologists. AMTS, Abbreviated Mental Test Score. Data for 2011 and 2023 include only partial years.

### Hip fracture incidence

**Figure 1:**
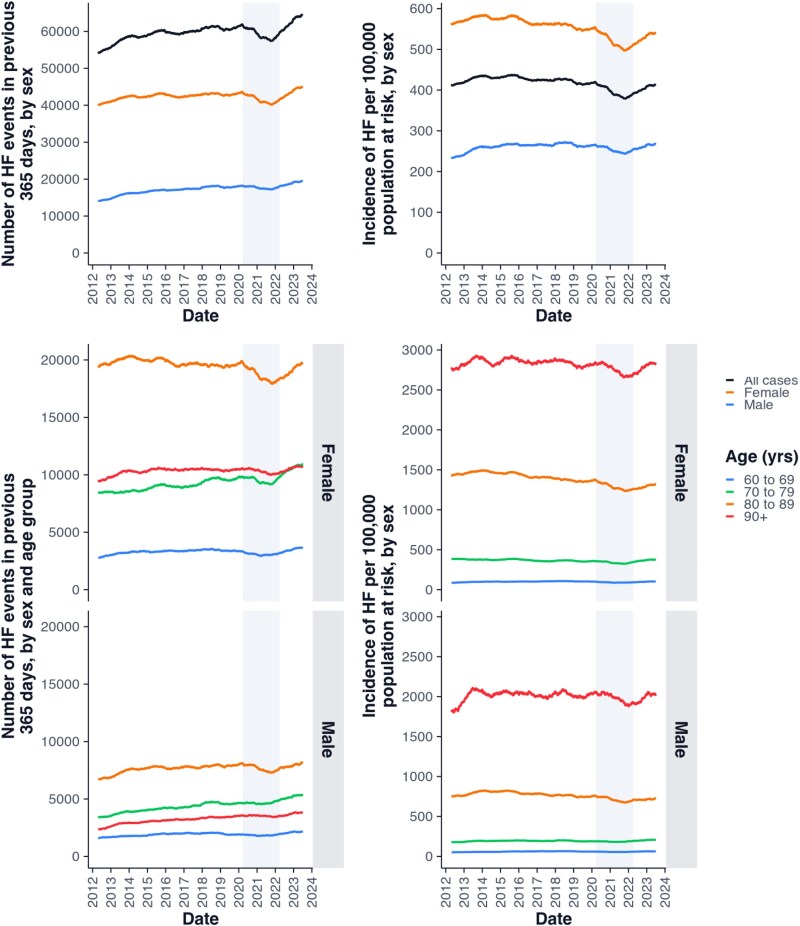
Hip fracture events and incidence over time by sex and age group. Figure 1—number (left column) of and incidence (right column) of HF events in the previous 365 days over time recorded in the NHFD. Incidence is derived using Office for National Statistics (ONS) mid-year population estimates for England, Wales and Northern Ireland and expressed per 100 000 of the at risk population for the given stratum with linear interpolation between mid-year estimates. The period of the COVID-19 pandemic is highlighted in grey.

Annual HF numbers increased from 55 832 (2012) to 63 341 (2022), representing a 13.5% rise in absolute case volume. However, overall incidence declined from 422.8 to 412.8 per 100 000 population between 2012 and 2022, indicating improved population-level fracture risk when accounting for demographic changes (Figure 1, Table 3). Despite declining overall incidence, absolute case numbers increased due to demographic shifts towards older age groups with inherently higher fracture risk.

Female incidence remained consistently more than double that of males throughout the study period. Sex-stratified analysis demonstrated divergent patterns: male incidence increased from 242.9 to 267.7 per 100 000, while female incidence declined from 574.9 to 539.8 per 100 000 between 2012 and 2022. Absolute case numbers showed growth in both sexes: females increased from 41 136 (2012) to 44 172 (2022) annually and males from 14 696 (2012) to 19 169 (2022) annually, with a notable transient decline in 2021 (males: 17 548; females: 40 644) coinciding with the COVID-19 pandemic.

Age-stratified incidence was highest in patients aged ≥90 years across both sexes. Among females, incidence in this oldest cohort declined 1.8% from 2855.0 per 100 000 to 2804.3 per 100 000 between 2012 and 2022. Male incidence in the ≥90 age group rose from 1988.8 (2012) to 2049.6 per 100 000 (2022), with a notable trough following the pandemic (1922.1 in 2021). Over the same period, the 80–89 year age group demonstrated declining incidence in both females (1451.5 to 1317.5 per 100 000) and males (768.5 to 723.4 per 100 000). In the 70–79 year cohort, female incidence decreased from 388.2 to 373.6 per 100 000, while male incidence increased from 183.7 to 207.7 per 100 000. Incidence in the 60–69 year cohort remained below 110 per 100 000 throughout (females: 92.8 to 101.8 per 100 000; males: 54.2 to 64.6 per 100 000), representing less than 10% of the total HF burden. The proportion of male patients increased progressively from 26.3% (2012) to 30.3% (2022), driven by increasing absolute male case numbers in all age groups.

Following the pandemic-related nadir in HF incidence, in 2020 and 2021, data to date suggest a reversal of the long-term declining trend in overall incidence. Between 2021 and 2022, overall incidence increased from 386.4 to 412.8 per 100 000, returning to near pre-pandemic levels. This pattern was observed across both sexes and all age groups.

**Table 3:** Hip fracture incidence per 100 000 in England, Wales and Northern Ireland.

								Year						
Group			Variable	2012	2013	2014	2015	2016	2017	2018	2019	2020	2021	2022
All			HF events	55 832	58 770	58 962	59 783	59 844	60 808	60 766	61 449	59 568	58 192	63 341
			Population at risk	13 205 188	13 382 714	13 592 237	13 748 788	13 943 367	14 144 337	14 354 909	14 606 016	14 804 165	15 060 594	15 342 889
			Incidence	422.8	439.1	433.8	434.8	429.2	429.9	423.3	420.7	402.4	386.4	412.8
By sex	Male		HF events	14 696	16 262	16 583	17 001	17 345	17 792	17 876	18 116	17 751	17 548	19 169
			Population at risk	6 050 067	6 149 746	6 260 382	6 349 464	6 453 131	6 560 022	6 671 126	6 798 268	6 897 132	7 021 484	7 160 119
			Incidence	242.9	264.4	264.9	267.8	268.8	271.2	268	266.5	257.4	249.9	267.7
	Female		HF events	41 136	42 508	42 379	42 782	42 499	43 016	42 890	43 333	41 817	40 644	44 172
			Population at risk	7 155 121	7 232 968	7 331 855	7 399 324	7 490 236	7 584 315	7 683 783	7 807 748	7 907 033	8 039 110	8 182 770
			Incidence	574.9	587.7	578	578.2	567.4	567.2	558.2	555	528.9	505.6	539.8
By sex and age	Male	60 to 69	HF events	1684	1795	1821	1971	2016	2029	2011	1918	1798	1883	2149
			Population at risk	3 106 079	3 131 059	3 142 603	3 154 130	3 172 762	3 124 348	3 121 053	3 145 051	3 184 551	3 247 851	3 326 974
			Incidence	54.2	57.3	57.9	62.5	63.5	64.9	64.4	61	56.5	58	64.6
		70 to 79	HF events	3522	3922	4042	4222	4295	4566	4585	4645	4592	4796	5303
			Population at risk	1 917 285	1 968 926	2 036 847	2 089 590	2 143 748	2 268 875	2 354 766	2 421 217	2 468 942	2 520 559	2 552 938
			Incidence	183.7	199.2	198.4	202	200.4	201.2	194.7	191.8	186	190.3	207.7
		80 to 89	HF events	6883	7590	7701	7684	7821	7820	7900	8015	7772	7432	7922
			Population at risk	895 616	912 540	935 232	955 445	979 612	1 004 633	1 029 716	1 058 993	1 069 477	1 074 263	1 095 052
			Incidence	768.5	831.7	823.4	804.2	798.4	778.4	767.2	756.9	726.7	691.8	723.4
		90+	HF events	2607	2955	3019	3124	3213	3377	3380	3538	3589	3437	3795
			Population at risk	131 087	137 221	145 700	150 299	157 009	162 166	165 591	173 007	174 162	178 811	185 155
			Incidence	1988.80	2153.50	2072.10	2078.50	2046.40	2082.40	2041.20	2045.00	2060.70	1922.10	2049.60
	Female	60 to 69	HF events	3016	3257	3323	3350	3379	3488	3418	3353	3050	3104	3543
			Population at risk	3 251 060	3 277 673	3 293 266	3 306 467	3 325 974	3 275 558	3 268 675	3 291 123	3 330 542	3 397 878	3 479 338
			Incidence	92.8	99.4	100.9	101.3	101.6	106.5	104.6	101.9	91.6	91.4	101.8
		70 to 79	HF events	8540	8525	8777	9089	8972	9367	9617	9842	9489	9409	10 682
			Population at risk	2 199 670	2 246 147	2 309 335	2 360 201	2 413 088	2 542 035	2 633 361	2 703 660	2 760 731	2 820 711	2 859 030
			Incidence	388.2	379.5	380.1	385.1	371.8	368.5	365.2	364	343.7	333.6	373.6
		80 to 89	HF events	19 734	20 366	19 949	19 892	19 692	19 583	19 468	19 673	18 847	18 044	19 297
			Population at risk	1 359 527	1 358 271	1 366 169	1 370 466	1 383 849	1 399 271	1 416 716	1 439 819	1 444 714	1 445 795	1 464 623
			Incidence	1451.50	1499.40	1460.20	1451.50	1423.00	1399.50	1374.20	1366.40	1304.50	1248.00	1317.50
		90+	HF events	9846	10 360	10 330	10 451	10 456	10 578	10 387	10 465	10 431	10 087	10 650
			Population at risk	344 864	350 877	363 085	362 190	367 325	367 451	365 031	373 146	371 046	374 726	379 779
			Incidence	2855.00	2952.60	2845.10	2885.50	2846.50	2878.80	2845.50	2804.50	2811.20	2691.80	2804.30

Number of hip fracture events and incidence by year in England, Wales and Northern Ireland. Population at risk is shown for age & sex strata, derived from Office for National Statistics (ONS) mid-year population estimates as at 30 June for each year. Incidence is reported per 100 000 of population at risk. Data for 2011 and 2023 are not shown as these include only partial years. HF, Hip fracture.

### Temporal and seasonal variation

Prior to March 2020, clear seasonal variation in HF incidence was observed, with winter peaks ~10–20% higher than summer troughs (see figure Appendix 2 in supplementary materials). Analysis of mean daily incidence demonstrated consistent cyclical patterns across all years from 2012 to 2019, with peak incidence occurring in winter months (December to February) and nadirs in summer (June to August). This pattern was most pronounced in patients aged ≥80 years and among female patients, whose baseline incidence across all age groups remained consistently higher than males throughout (~1.6 vs 0.7 per 100 000 population per day). In females, age-stratified analysis revealed incidence rates of ~8 per 100 000 per day in the ≥90 year cohort, compared with 4 per 100 000 in 80–89 year-olds and less than one per 100 000 per day in 60–69 year-olds, with these relative differences maintained consistently across the study period.

The beginning of the COVID-19 pandemic and national lockdown (March 2020) resulted in an acute transient decline in daily presentations, most evident in the overall incidence time series and particularly pronounced among patients aged 80–89 years. This disruption in the seasonal pattern persisted throughout 2020 and into 2021, with notable flattening of the expected winter peak in late 2020. Incidence returned gradually to pre-pandemic levels by early 2022, with re-emergence of seasonal variation, albeit with attenuated amplitude compared with pre-pandemic years. By 2023, seasonal cyclicity appeared to have been re-established.

### Physical status trends

**Figure 2:**
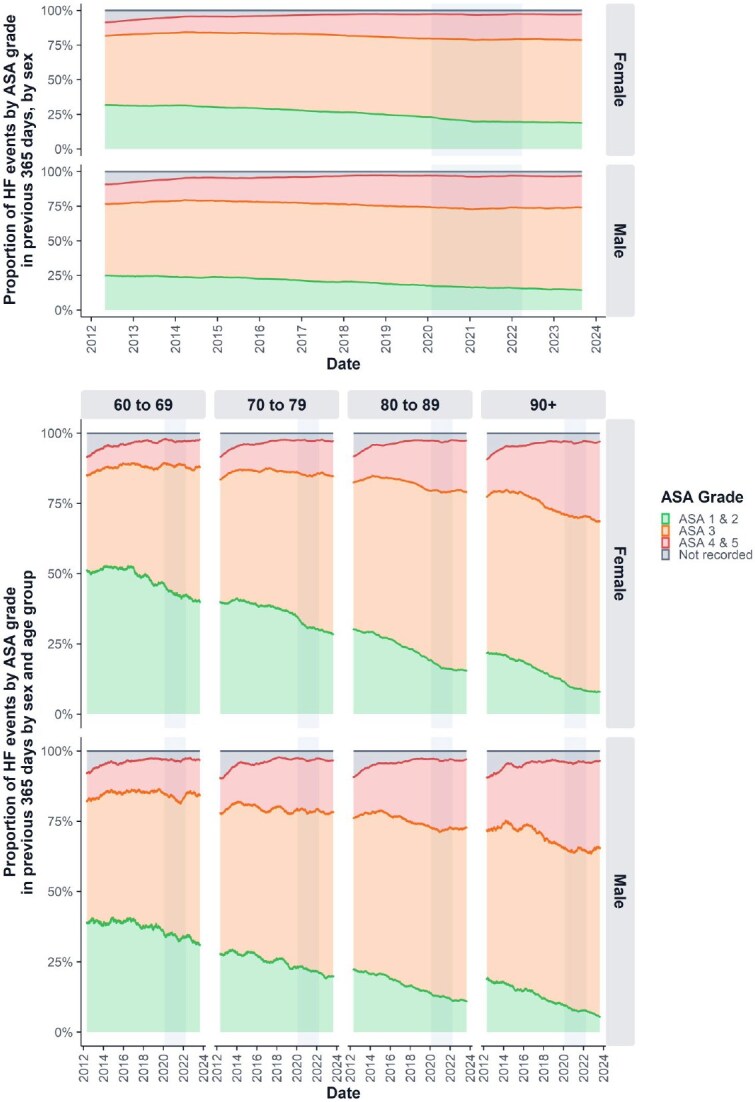
Hip fracture events by ASA grade over time. Figure 2—stacked percentage plots showing proportion of HF events represented each ASA grade group by sex (top chart) and by sex and age group strata (bottom chart) in previous 365 days over time. The period of the COVID-19 pandemic is highlighted in grey.

A progressive increase in patients with severe systemic disease was observed across both sexes (Figure 2). Among females, the proportion of cases recorded as ASA ≥3 increased from 66.4% (2012) to 80.4% (2022), and from 73.4% to 84.3% among males; over the same period, the proportion classified as ASA 4–5 approximately doubled, indicating increasing multimorbidity and case complexity.

Age-stratified analysis revealed differential patterns of progression. In the 60–69 year cohort, the proportion of cases recorded as ASA 1–2 declined from ~50% to 40% (females) and 50% to 35% (males) between 2012 and 2023, with corresponding increases in ASA 3 and ASA 4–5 classifications. Similar trends were observed across all sex and age groups, with the most marked shift occurring in older age groups. Among patients aged 80–89 years, the proportion of cases recorded as ASA 1–2 decreased from 32% to 15% (females) and 28% to 10% (males). In the oldest cohort (≥90 years), ASA 1–2 represented less than 25% of female patients and less than 20% of male patients by 2012, declining further to ~10% and 5% respectively by 2023. Conversely, ASA 4–5 increased from ~5% to 10% across most age groups, with the steepest rise observed in patients aged 80–89 years.

Despite this trend in poorer physical status, an increasing proportion of patients were admitted from their own home or sheltered housing rather than institutional care, and fewer were housebound prior to fracture (Tables 1 and 2). This has implications for discharge planning, as services must now support the return of increasingly complex patients to community settings, placing greater demands on intermediate care and home-based support services.

## Discussion

This study provides contemporary evidence that, despite the general rise in annual HF numbers, overall incidence has fallen in women who comprise the majority of presentations. We observed three key epidemiological shifts: first, substantial sex divergence is occurring—with male incidence rising whilst female incidence falls, resulting in males now making up nearly one-third of all HFs. Second, the COVID-19 pandemic caused acute disruption to HF presentations in early 2020, followed by gradual recovery through 2021–2022, with evidence of rising incidence now in both men and women in the most recent data; whether this represents a sustained trend reversal or transient recovery from pandemic-related suppression remains to be seen given the limited post-pandemic observation period. Third, patient complexity has increased markedly, with the proportion presenting with severe systemic disease (ASA ≥3) rising to over 80% of cases, with the ASA 4–5 subgroup approximately doubling. The burden remains heavily concentrated in the oldest age groups, though younger old-age cohorts demonstrate concerning rises in both incidence and comorbidity burden.

These above multifactorial trends indicate that whilst population-level fracture risk improved modestly over the decade (particularly for females), HF patients are now older, more commonly male and considerably more medically complex than previously, with implications for health service resources and clinical outcomes.

The declining incidence observed in this study has been noted in England, and a number of high-income countries, including the USA, Scandinavia and other parts of Europe [1]. Most recently, Webster et al used Hospital Episode Statistics for England to report broadly similar temporal patterns in HF epidemiology; the study included adults aged ≥50 years and found a modest pre-pandemic decline in overall incidence, a pandemic-related fall to ∼4% below expected levels, and subsequent post-pandemic rates around 3% above those expected. These results are highly concordant with our NHFD-based findings of falling pre-pandemic incidence, an acute nadir in 2020–2021, and subsequent rebound to near pre-pandemic levels. Both studies demonstrate persistent female predominance but a rising absolute burden and proportion of male cases and increasing HF rates in older age groups in the post-pandemic period. Important methodological differences exist; our analysis spans England, Wales and Northern Ireland, uses a higher age threshold (≥60 years), and leverages the clinically rich NHFD to describe trends in comorbidity.

Earlier research focussing on temporal trends before 2018 has attributed the declining incidence in part to improved lifestyle, such as reducing prevalence of smoking [10] and alcohol [11], increasing body mass index (BMI) [12], the use of calcium and vitamin D supplementation [13, 14] and osteoporosis treatment [15]. Since then there have been updated national guidelines [16] as well as ‘major improvements in the identification of patients and falls assessment’ [17]. However, these improvements are not seen in all cohorts, with the incidence of HFs rising in males. This sexual disparity has been noted previously with males having between 29.8% and 66.5% lower use of anti-osteoporosis medication when compared to females [1]. It has been postulated that this might be due to postmenopausal women being treated for primary prevention of osteoporotic fractures, whilst males are treated for secondary prevention once they have had a fracture [1]. More recently, this trend has also been demonstrated in the Scottish cohort of HFs [2]. Importantly, male HFs also represent a cohort with poorer outcomes; and a higher mortality rate [1], lower likelihood of post-fracture independent mobility and/or return to their own home [18].

The COVID-19 pandemic impacted HF incidence, with periods of acute decrease, gradual recovery and subsequent rise in incidence. It has been previously shown that whilst on a population level, falls and fracture rates were below the predicted for the period of lockdown, people living with frailty had a higher falls and fracture rate [19], suggesting that these observed temporal changes may not be due to the effect of lockdown. An alternative explanation for the temporary reduction in HF incidence during 2020–2021 is mortality displacement within the general population. The COVID-19 pandemic disproportionately increased mortality among frail older adults with multiple comorbidities—the same population at highest risk of HF. [7, 20] It is plausible that a proportion of individuals who would otherwise have sustained an HF in subsequent years died during the pandemic, effectively removing them from the population at-risk of sustaining an HF. The subsequent rise in incidence from 2022 onwards may therefore represent replenishment of the ‘at-risk’ patient population; this hypothesis and time-frame is consistent with previous modelling by our group showing that deaths among individuals who sustained HFs during the COVID-19 pandemic were displaced (brought forward) by between 135 and 535 days on average. [21] Assuming similar effects in the general population, if mortality displacement contributed significantly to the observed reduction in HF incidence, its influence would be expected to attenuate in the coming years. This makes interpretation of temporal trends during and immediately after the pandemic period challenging, and caution is warranted when using even recent data for performance benchmarking or service planning.

Patients sustaining HF are becoming increasingly comorbid and presenting with greater systemic disease, with a gradual and sustained increase in the proportion of cases categorised as ASA grade 3 or higher in both females and males. Data from the Danish Registry has shown that multimorbidity and HF surgery interact synergistically, increasing the risk of infections [22], myocardial infarction and stroke [23] as well as venous thromboembolism [24]. A higher comorbidity index is also associated with a higher mortality rate [25]. Males are more likely to be comorbid than females and this might be a contributing factor to their higher mortality rate seen in this cohort [1]. Data such as this further highlights the importance of an integrated geriatric and orthopaedic multi-disciplinary model. It is well established that the orthogeriatric model improves length of stay and functional outcomes and reduces complications [26–29] with early geriatric intervention important [30]. It is therefore paramount that, in a cohort that is increasingly complex, there is appropriate funding and resource allocation into this area.

Over 20% of all patients with HFs are at risk of malnourishment or are malnourished, and this increases to almost a third in female patients. Recently, improvements were seen in males, but not females. Malnourishment is associated with an increased risk of delirium, decline in mobility and independence and mortality [31] and therefore represents an important modifiable risk factor to address. Simple measures, such as oral nutritional supplementation, can decrease the risk of complications and reduce length of stay [32]. Hip fracture configuration has remained relatively static with the proportion of pertrochanteric, subtrochanteric and intracapsular neck of femur fractures comparable across the study period (Table 1 & 2). However, there has been an increasing proportion of displaced, compared to undisplaced, intracapsular neck of femur fractures. Osteoporosis progression has been previously shown to be correlated with more complicated acetabular fractures [33] and thus it could be inferred that osteoporotic HFs are more likely to be displaced. Similarly, increasing computed tomography (CT) scan utilisation [34] not only for diagnosis, but delineating between displaced and undisplaced fractures may have contributed to the increase.

The increasing proportion of patients presenting with severe systemic disease and high pre-fracture care needs also strengthens the case for consideration of non-operative, palliative treatment pathways in carefully selected patients. The FRAIL-HIP study demonstrated that, for frail institutionalised patients with limited life expectancy, non-operative management selected through a shared decision-making process yielded comparable health-related quality of life and high treatment satisfaction relative to surgery, with a humane dying process where death occurred [35, 36]. As case-mix in the UK shifts further, integration of structured shared decision-making and palliative pathways alongside the established orthogeriatric model may warrant greater attention.

We acknowledge several limitations. Although data completeness is high, some variables contained missing or unrecorded entries, which may introduce measurement bias, particularly for ASA and frailty-related metrics such as AMTS, nutrition (MUST) score. Second, the NHFD does not capture detailed comorbidity profiles, medication history, bone health assessments or mechanism of injury, which limits exploration of causal drivers behind the observed epidemiological shifts. Comorbidity profiles are of particular relevance as there are limitations to the use of ASA grading. ASA classification determines physical status and ‘fitness’ for an anaesthetic and thus, patients who have one or multiple severe systemic diseases are classified as the same grade, underappreciating case complexity. Other risk factors, such as sarcopenia and malnutrition, are independent predictors of poor outcomes [37] with only the latter included in the NHFD. Similarly, other scoring systems such as frailty scores [38], Nottingham HF scores and age adjusted comorbidity/Charlson index scores [39] are better at predicting some endpoints, such as mortality, and might therefore better reflect case complexity. Third, incidence calculations relied on mid-year ONS population estimates and the assumption of linear interpolation between years; as such, this approach may not fully account for rapid demographic fluctuations (whether seasonal or pandemic-related) in the underlying at-risk population. Fourth, the definition of the COVID-19 period, while transparent and consistent with national policy timelines, may not precisely reflect local variation in service disruption, testing access or infection burden. Finally, the most recent post-pandemic data available represent only a short duration follow-up window, restricting interpretation of whether the apparent rise in incidence reflects true reversal of long-term trends or transient recovery from pandemic-related suppression.

In conclusion, the falling overall incidence across England, Wales and Northern Ireland is likely multifactorial. However, annual HF numbers continue to rise, patients are now older, more commonly male, and substantially more comorbid. This worsening case load and complexity mandates urgent realignment of care resources to minimise the impact on patient mortality and economic health burden. Prevention strategies must extend beyond postmenopausal women to include older men, and acute services require capacity for increasingly complex perioperative care. Furthermore, with more complex patients now living independently prior to fracture, discharge planning and community-based intermediate care services face increasing pressure to return these individuals safely home. Finally, caution is needed when interpreting key performance indicators around the pandemic period due to potential confounding from mortality displacement.

## Supplementary Material

aa-26-0278-File004_afag228

appendix_1_afag228

appendix_2_afag228
