# Supplementary material for: A decade of increasing complexity in patients sustaining a hip fracture in England, Wales and Northern Ireland: a national cohort study using the National Hip Fracture Database dataset
Source: Age Ageing. 2026 Aug 11;55(8):afag228. doi: 10.1093/ageing/afag228 (PMC13461217; doi:10.1093/ageing/afag228)
Supplement: Supplementary file 1 [file aa-26-0278-file004_afag228.docx]

**A decade of increasing complexity in patients sustaining a hip fracture in England, Wales, and Northern Ireland: a national cohort study using the NHFD dataset**

**Supplementary Material**

**Contents**

Appendix 1. Study flow diagram

Appendix 2. Cyclical variation in hip fracture events and incidence by sex and age group

**Appendix 1. Study flow diagram**

**Appendix 1.** Study flow diagram. NHFD, National Hip Fracture Database.

**Appendix 2. Cyclical variation in hip fracture events and incidence by sex and age group**

**Appendix 2.** Plot matrix showing annual cyclical variation in hip fracture events. Top chart shows the mean daily hip fracture incidence per 100,000 of the given stratum’s population at risk for the previous 30 days by sex. Bottom chart shows the mean daily hip fracture incidence per 100,000 of the given stratum’s population at risk for the previous 30 days by sex and age group. The period of the COVID-19 pandemic is highlighted in grey. Vertical dashed lines indicate 1st January each calendar year. HF, Hip fracture.
