# Supplementary material for: A decade of increasing complexity in patients sustaining a hip fracture in England, Wales and Northern Ireland: a national cohort study using the National Hip Fracture Database dataset
Source: Age Ageing. 2026 Aug 11;55(8):afag228. doi: 10.1093/ageing/afag228 (PMC13461217; doi:10.1093/ageing/afag228)
Supplement: Supplementary file 2 [file appendix_1_afag228.docx]

**A decade of increasing complexity in patients sustaining a hip fracture in England, Wales, and Northern Ireland: a national cohort study using the NHFD dataset**

**Supplementary Material**

**Appendix 1. Study flow diagram**

**Appendix 1.** Study flow diagram. NHFD, National Hip Fracture Database.
